# Guarding Vibrations—*Axestotrigona ferruginea* Produces Vibrations When Encountering Non-Nestmates

**DOI:** 10.3390/insects12050395

**Published:** 2021-04-29

**Authors:** Kathrin Krausa, Felix A. Hager, Wolfgang H. Kirchner

**Affiliations:** 1 Behavioural Biology and Biology Education, Ruhr University Bochum, Universitätsstraße 150, 44780 Bochum, Germany; felix.hager@rub.de (F.A.H.); wolfgang.h.kirchner@rub.de (W.H.K.); 2 International Centre of Insect Physiology and Ecology (icipe), Nairobi P.O. Box 30772-00100, Kenya

**Keywords:** nest defense, biotremology, nestmate recognition, alarm, substrate-borne vibration

## Abstract

**Simple Summary:**

Stingless bees visit flowers to collect pollen and nectar which is stored in their nest. These stores are highly valuable and help the colony to overcome times of resource scarcity. The bees protect the collected nectar and pollen against robbers of all kind. It should be advantageous for a colony if it can recognize intruders and chase them away. In this context, the communication with nestmates might be crucial. The most important signals that stingless bees use are chemicals and vibrations. Communication between nestmates has been mostly studied in the context of foraging. Little is known about communication in the context of defense. We tested if vibrational signals play a role in nest defense and nestmate recognition of the African stingless bee *Axestotrigona ferruginea*. Stingless bees produce distinct vibrations in the context of foraging and guarding. Foraging vibrations most likely contain food source information whereas guarding vibrations might be used to alarm nestmates.

**Abstract:**

Flower visiting stingless bees store collected pollen and nectar for times of scarcity. This stored food is of high value for the colony and should be protected against con- and heterospecifics that might rob them. There should be high selective pressure on the evolution of mechanisms to discriminate nestmates from non-nestmates and to defend the nest, i.e., resources against intruders. Multimodal communication systems, i.e., a communication system that includes more than one sensory modality and provide redundant information, should be more reliable than unimodal systems. Besides olfactory signals, vibrational signals could be used to alert nestmates. This study tests the hypothesis that the vibrational communication mode plays a role in nest defense and nestmate recognition of *Axestotrigona ferruginea*. Substrate vibrations induced by bees were measured at different positions of the nest. The experiments show that guarding vibrations produced in the entrance differ in their temporal structure from foraging vibrations produced inside the nest. We show that guarding vibrations are produced during non-nestmate encounters rather than nestmate encounters. This further supports the idea that guarding vibrations are a component of nest defense and alarm communication. We discuss to whom the vibrations are addressed, and what their message and meaning are.

## 1. Introduction

Stingless bees (Meliponini) comprise more than 500 different species that show a remarkable diversity of life histories and ecological adaptations [1]. The highly eusocial stingless bees (Meliponini) belong to the Apidae and are a sister-tribe to the highly eusocial honeybees (Apini), the eusocial bumblebees (Bombini) and the mostly solitary orchid bees (Euglossini) [2].

Like in all eusocial insects, dozens to thousands of individuals live together. For the colony’s success, individual behavior must be coordinated, and therefore the ability to communicate is crucial. Communication has been primarily studied in the context of foraging. Foragers of some species guide nestmates by leaving scent marks at and on the way to food sources (for review see [3]). In other species, successful foragers that return to the hive produce thoracic vibrations (tremulations sensu [4]) that are modulated, depending on the food source’s profitability [5,6]. There is much evidence showing that stingless bees use these chemical and mechanical pathways in a complex multimodal communication system [7].

Stingless bees store collected pollen and nectar in wax pots to be prepared for times of scarcity. The stored food is highly valuable to the colony and attractive for hetero- and con-specifics. Honey and pollen might be robbed by humans, apes, ants, beetles, phorid flies as well as other bees, including stingless bees [8,9,10,11]. Robbery by conspecifics might occur if the profit of a raid is higher than its expected costs. This should apply whenever floral resources are scarce and/or the putative victims of the robbery cannot appropriately defend themselves. Sophisticated defensive strategies that include non-nestmate identification, alarm communication and fighting are crucial for the colony’s success and sometimes survival [11].

Nestmate recognition in stingless bees is mediated mainly by chemical cues [12,13,14]. Some species carry cuticular hydrocarbons that have a unique profile containing information about the species, its genetic lineage, caste, sex, reproductive status, function in the colony, as well as nest origin [15,16].

When individuals meet, they compare the interaction partner’s specific odor with their own olfactory background. In the Neotropical stingless bee *Frieseomelitta varia* it was shown that incoming conspecific bees are only accepted if their odor profile lacks undesirable components despite the presence of desirable cues [17]. If the odor profile of an individual matches the template, it is allowed to enter the nest. In contrast, an odor profile that does not match the template can cause agonistic behavioral reactions. Individuals are chased or mandibles are used to fight against the intruder (for review see [11]).

To reinforce the defense, it would be advantageous if nestmates get informed about an attack. In this context, social insects often make use of chemical signals [13,18]. Stingless bees are known to use alarm pheromones that originate from the mandibular glands. If released, they elicit defensive, i.e., aggressive behavior [14,19,20,21].

In addition to chemical signals, some social insects employ mechanical signals in nest defense (for review see [22,23,24]). Bumblebees produce buzzing and hissing sounds when being disturbed [25,26,27,28]. Hissing appears to be an aposematic alarm signal addressed to predators entering the nest [28]. Buzzing sounds are associated with nest defense and are believed to serve to communicate with nestmates [25,29]. Hissing sounds are also emitted by some honeybee species [30,31,32,33,34,35,36,37,38,39,40]. As in bumblebees, hissing is believed to be primarily an aposematic alarm signal. If nests are disturbed by an approaching human, foragers might emit an initial piping sound upon arrival in the colony. This is followed by hissing sounds produced by a large number of bees. Individuals standing close to the piping bee will start hissing and neighboring bees join in. This leads to rapid propagation of the signal [36]. Piping and hissing leads to a decrease in worker activities such as forager dancing and departures from the colony [36]. Most studies recorded the airborne sound that is emitted by bees. However, in some cases, it turned out that substrate-borne vibrations rather than sound are crucial for signal perception. Substrate-borne vibrations have many peculiarities when compared to sound vibrations [41,42]. The field of biotremology has only recently begun to diverge from bioacoustics to address the study of the vibrational communication mode.

Until now it is not clear whether vibrations play a role in stingless bee nest defense. Smith and Roubik [19] found *Melipona* bees respond to mandibular gland extracts with alarm recruitment and defensive behavior. Workers fly out of the nest, land on an intruding object, and vibrate their flight muscles during biting. Other studies report that bees produce intense buzzing sounds in the presence of alarm pheromones [21,43,44]. This buzzing, however, has not been studied any further. *Axestotrigona ferruginea* is known to use vibrational signals that are believed to contain foraging-related information [45,46]. The present study asks whether *A. ferruginea* makes use of vibrational signals in the context of nest defense. For efficient communication, the vibrations produced in the foraging context should differ from those produced in the context of nest defense. If *A. ferruginea* makes use of distinct vibrations in the context of nest defense, they should be produced more often during non-nestmate encounters compared to nestmate encounters. To test this hypothesis, we performed controlled nestmate recognition experiments.

## 2. Materials and Methods

### 2.1. Study Site and Species

The study was carried out during the dry season from June to July 2015 at the Goro Research Camp in the Soutpansberg Mountain Range in South Africa (22°56′04.9 S, 29°25′49.4 E). The Soutpansberg Mountains are one of South Africa’s biodiversity hotspots [47]. At least eight flower-visiting stingless bee species (*A. ferruginea* Lepeletier 1961, *Meliplebeia beccarii* Moure 1961, *Plebeina armata* Friese 1900, *Hypotrigona araujo* Michener 1959, *Hypotrigona gribodoi* Magretti 1884, *Hypotrigona ruspolii* Magretti 1898, *Liotrigona bottegoi* Magretti 1895, *Liotrigona parvula* Darchen 1971) and one obligate cleptoparasitic species (*Cleptotrigona cubiceps* Moure 1961) can be found there [1,48]. For the experiments, four nests of *A. ferruginea* were used. Colonies were in the crevices of a stone wall and a hollowed trunk of *Euphorbia ingens* E. Mey. ex Boiss. Experiments were conducted between 10:00 a.m. and 4:00 p.m. in the shade. The ambient temperature ranged during experimentation from 16–23 °C (measurements from a local weather station, in vivo).

### 2.2. Vibration Recordings

Substrate vibrations were measured at different positions of the nest using accelerometers (Metra; KD37, ±3 dB in the range from 1 Hz to 15 kHz; KS95B.100, ±3 dB in the range from 1 Hz to 15 kHz; Radebeul, Germany), a charge amplifier (Metra M68D1, Radebeul, Germany) and a digital audio recorder (96 kHz, 32-bit wav, Tascam DR-40, Wiesbaden, Germany).

#### 2.2.1. In the Nest

To measure vibrations produced in the nest, an accelerometer (Metra, KD37, ±3 dB in the range from 1 Hz to 15 kHz, Radebeul, Germany) was mounted on the nest structure 10 cm below the nest entrance tube. Bees’ wax was used to ensure a tight coupling of the accelerometer to the nest structure. Wax was attached to the nest at least two days prior to experimentation to ensure adaptation to putative chemical cues. Substrate vibrations were measured continuously in four time intervals of 10 min each while bees were foraging, i.e., flying undisturbed in and out of the nest. After vibration recordings, the nest was opened at the place of the accelerometer attachment. Careful probing revealed honeypots in the hollow behind the nest surface.

#### 2.2.2. At the Entrance Tube

Due to the small and fragile entrance tube, a lightweight IEPE accelerometer (Metra, KS95B.100, ±3 dB in the range from 1 Hz to 15 kHz, Radebeul, Germany) was used to measure vibrations produced in the nest entrance. Bees’ wax was used to couple the accelerometer to the entrance tunnel. The production of vibrations was measured continuously in four intervals of 10 min each. Before these recordings, guards of a foreign nest of *A. ferruginea* were restrained in a 1.5 mL Eppendorf vial and pressed to bite in a piece of filter paper. In doing so, the bees released an amount of about 1 µL of liquid, presumably mandibular gland secretion on the filter paper. The filter paper was placed in a 1.5 mL Eppendorf vial and stored for a maximum of 30 min. The vial was opened and carefully mounted 1 cm next from the entrance tube using bees’ wax in the same way as mentioned above. Immediately after mounting, we recorded substrate vibrations.

#### 2.2.3. In the Arena

Pairs of worker nestmates (*n* = 26) or non-nestmates (*n* = 28) were introduced in Petri dishes (Ø 3.5 cm, henceforth arena). We used Sellotape to tightly couple the dish’s bottom and lid. The arenas could be entered through an opening that was drilled in the lid and could be closed using Sellotape. Bees from four different nests were caught in 1.5 mL Eppendorf vials and kept separately until used for experiments. Bees guarding in the nest entrance were caught using an aspirator. All bees carried wax on their hind legs as it is described for guards in other species [49,50,51]. Bees were stored for a maximum of 30 min. The vials containing a bee were opened and placed above the arena’s opening. One after the other either nestmates or non-nestmates walked from the vial into the arena. The arena was centrally mounted on top of an accelerometer (Metra, KD37, ±3 dB in the range from 1 Hz to 15 kHz; Radebeul, Germany) using double-faced adhesive tape. Vibration recordings were synchronized with video recordings using the audio output of the digital audio recorder (Tascam DR-40, Wiesbaden, Germany) as audio input for the video camera (Panasonic, HC-V720). The bees were filmed to ensure a subsequent blind analysis of their behavior, i.e., to ensure that the observer was not aware of whether nestmates or non-nestmates were observed. Following Kirchner and Friebe [52], we observed and quantified the total number of antennation, biting and escape responses for 5 min, beginning when the second bee entered the arena. We applied a generalized linear mixed model based on a log-linear Poisson distribution (SPSS 27). Nestmates/non-nestmates were included as a fixed effect term and the individual combination with respect to the bee’s nest origin as a random effect term. Response variables were antennation, escape response, biting and pulses.

### 2.3. Vibration Analysis

**Figure 1:**
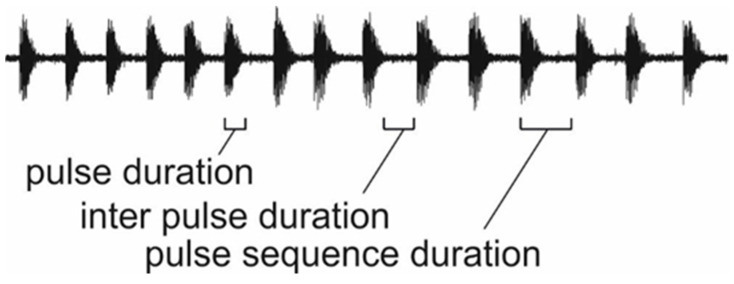
Temporal structure of vibrational pulse trains produced by *A. ferruginea*.

We compared the temporal structure of vibrational pulse trains produced in the nest, in the entrance tube and in the arena. A pulse train is defined as at least three consecutive pulses that were separated by less than 2 s. Pulse duration was measured from spectrograms (Hanning, 1024 samples). This allowed us to clearly identify the on- and offset of vibrations despite low-frequency noise. The following temporal characteristics were analyzed: pulse sequence duration, pulse duration, inter-pulse duration and duty cycle (pulse duration/pulse sequence duration) (Figure 1). To avoid pseudo-replication, we averaged a repetitive pattern per pulse train.

As a measure of the variability of the pulse trains’ temporal structure, we calculated the coefficient of variation (CV = SD × 100/mean). CV was compared using the Kruskal-Wallis test since data were not normally distributed. For pairwise comparison, Dunn-Bonferroni was used as a post-hoc test (SPSS 25). Audio files were bandpass filtered (0.1–3 kHz) to match a fundamental frequency of around 300 Hz and harmonics. Files were analyzed manually and blind, with the experimenter unaware of the recording location (Raven Pro 1.4).

## 3. Results

### 3.1. Production of Vibrations at the Nest

**Figure 2:**
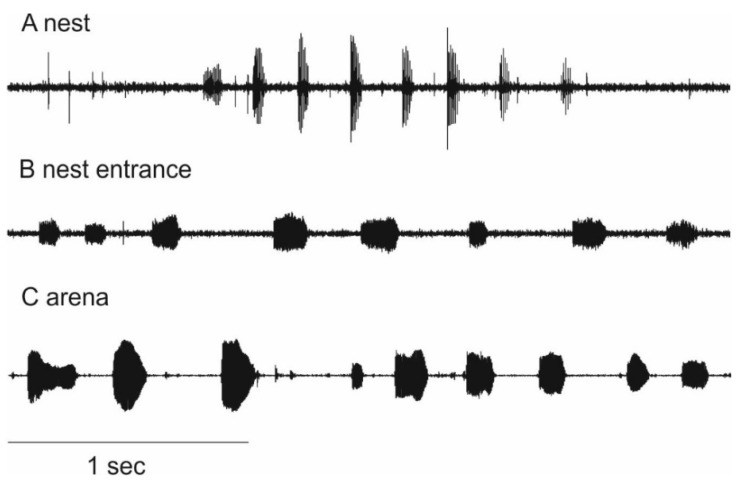
Vibrational pulse trains produced by *A. ferruginea* at different measurement points (bandpass filter 0.1–3 kHz) where (**A**) is Foraging vibrations produced inside of the nest, (**B**) is Guarding vibrations produced in the entrance tube, and (**C**) is Guarding vibrations produced in an arena.

Vibrations produced at different locations at the nest differ considerably. Details on the temporal structure of pulse trains are given in Table 1. Thoracic vibrations produced in the nest were measured while bees were foraging. The temporal structure of these vibrations is relatively uniform (Figure 2A). This is indicated by a low relative standard deviation of the pulse trains’ temporal structure. Pulse sequence duration, for example, varies only by 23.7% (Table 2).

Vibrational pulse trains produced in the entrance tube were recorded after the attachment of a vial containing alarm pheromone. During these experiments, some bees retreated in the nest, while others flew out of the nest, hovered in front of it and attacked the vial. Vibrational pulse trains produced in the entrance tube are less uniform than those produced in the nest (Figure 2B). Coefficient of variation (CV) of pulse sequence duration, inter-pulse duration and duty cycle of vibrations produced in the entrance tube are significantly different from those produced in the nest (Table 2).

### 3.2. Arena Experiments

**Figure 3:**
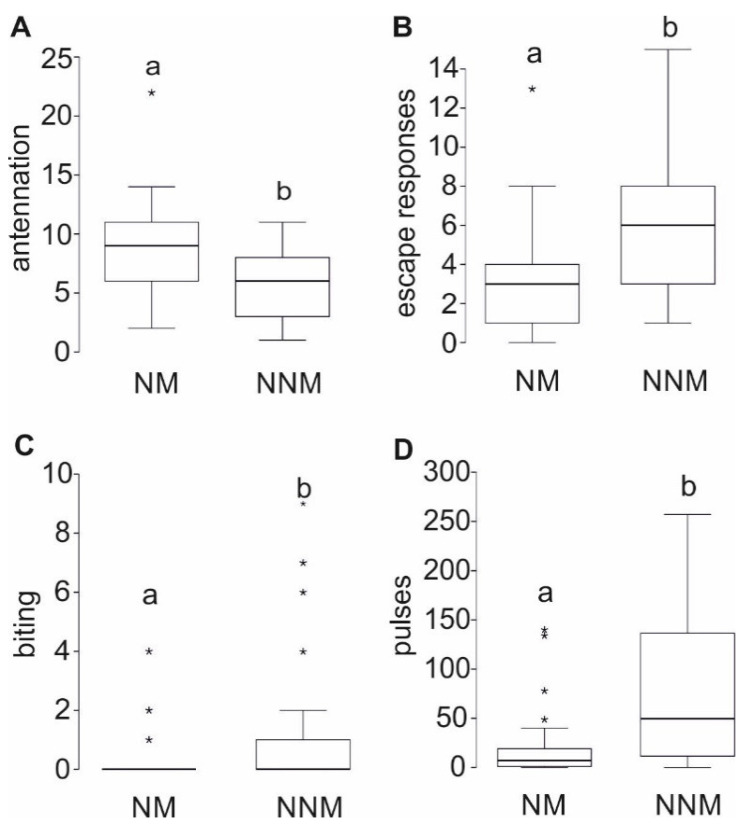
Interaction between nestmates (NM) and non-nestmates (NNM) of *A. ferruginea* during 5-min arena experiments. (**A**) Nestmates have significantly more antennal contacts than non-nestmates (F = 33.436; *p* < 0.001). (**B**) Non-nestmates showed significantly more escape responses than nestmates (F = 6.576; *p* = 0.013) (**C**) Non-nestmate performed significantly more biting attacks than nestmates (F = 5.743; *p* = 0.02) (**D**) Non-nestmates produce significantly more vibrational pulses than nestmates (F = 6.597; *p* = 0.013)Boxplots show median (thick horizontal line), upper and lower quartile (box), 1.5× inter quartile range (whiskers) and outliers (asterisks). Different letters indicate significant differences. Sample size: nestmates = 26, non-nestmates = 28.

The behavior of non-nestmate (NNM, *n* = 28) and nestmate (NM, *n* = 26) pairs of four *A. ferruginea* nests was observed. The generalized linear mixed model revealed that the combinations of individuals, i.e., the individuals’ origin does not have an influence on the observed variability (antennations: Z = 1.177, *p* = 0.239; escape responses: Z = 1.206, *p* = 0.228; biting: Z = 1.349, *p* = 0.177; pulses: Z = 1.922, *p* = 0.055). In contrast, the model revealed that the bees discriminate nestmates from non-nestmates. Antennations are performed more often in nestmate than non-nestmate encounters (Figure 3A: F = 6.597; *p* = 0.013). Escape responses and biting attacks occur significantly more often in non-nestmate pairs than nestmate pairs (Figure 3B,C: F = 6.576; *p* = 0.013 resp. F = 5.743; *p* = 0.02).

During arena experiments, bees produce vibrational pulse trains (Figure 2C). The coefficient of variation of the vibrations’ temporal structure produced in the arenas does not differ from those produced in the entrance tube but differs from those produced in the nest (Table 2). Non-nestmates produce significantly more vibrational pulses than nestmates (Figure 3D: F = 33.436; *p* < 0.001). Bees neither have direct body contact during pulse production nor can any distinct movement or behavior like wing fanning be associated with the vibrations.

## 4. Discussion

Stingless bees live in perennial nests where they store resources that are highly valuable for the colony and are attractive for an array of con- and heterospecific non-nestmates. Plenty of species are known to take advantage of stingless bee food stores [8,9,10,11]. Some stingless bee species are obligate cleptoparasites. They solely rob nests of other stingless bee species and do not visit flowers themselves [53]. It is believed that the defensive repertoire of species was shaped under the selective pressure of parasites and predators [54].

If an intruder approaches the nest entrance, the bees’ primarily need is to discriminate it from nestmates and determine the adequate response action. If a conspecific intruder tries to enter the colony, the discrimination task is not trivial. However, several stingless bee species are able to discriminate between nestmates and non-nestmates [52,55,56,57,58,59,60,61,62]. Our experiments reveal that *A. ferruginea* is no exception. In arena experiments, *A. ferruginea* discriminate nestmates from non-nestmates and non-nestmates attack each other more often than nestmates.

An efficient alarm communication would considerably contribute to a successful defense of the nest. We hypothesized that *A. ferruginea* makes use of vibrations in the context of nest defense and assumed that these vibrations are different compared to those produced in a foraging context. There are indications that vibrations in the nest are modulated signals produced by foragers and used to communicate food source information [5,6]. The signals’ pulse sequence duration, pulse duration, inter-pulse duration and duty cycle are expected to vary between signals, depending on food source characteristics. A study on *A. ferruginea*, revealed that the intra-signal variability of these patterns is lower when compared to inter-signal variability [46]. In other words: the temporal pattern, e.g., pulse duration of different vibrational signals, is variable and might be due to different information encoded, whereas variability within a signal is lower. This relatively low intra-signal variability is thought to be due to a redundant repetition of information encoded in the signal’s temporal structure. The coefficient of variation is a measure of intra-signal variability. In this study, we found the variability of vibrational pulse trains produced in the nest to be in line with a previous study [46] and henceforth refer to them as foraging vibrations. We expect the intra-signal variability between individual bees performing different tasks greater than the averaged variability between different nests, therefore, we did not test for inter-colonial differences. With a bigger data set, it would be interesting to analyze colony-specific signaling behavior.

Vibrational pulse trains recorded in the entrance tube were produced in a different context than foraging. Bees standing in the entrance tube were confronted with an alarm pheromone. In keeping with other studies [19,21,44], it can be assumed that the alarm pheromone at the entrance tube is a threat, which elicits alarming behavior. In *A. ferruginea* this was indicated by the fast retreat of most bees into the nest. Besides that, some bees performed hovering flights and attacked the filter paper carrying the alarm pheromone. The intra-signal variability of vibrational pulse trains produced in this context is higher compared to the intra-signal variability of foraging vibrations. We propose to name vibrations produced in this context “guarding vibrations”. We performed nestmate recognition experiments under controlled conditions in arenas. It can be assumed that a non-nestmate in the arena is considered as a threat to the bee, consequently, we expected them to produce vibrational pulse trains during non-nestmate encounters. This is exactly what we found; the bees produce vibrations more often when encountering non-nestmates compared to nestmates. Furthermore, the vibrational pulse trains produced in the arena have a similar temporal structure as guarding vibrations and should be referred to in the same way.

### Vibrational Signaling?

In animal communication studies, a fundamental distinction between signals and cues is made. Signals have evolved specifically to alter a receiver’s behavior whilst cues are incidental sources of information detected by unintended receivers [63,64]. So far, the information on whether guarding vibrations are signals or cues is not certain since it is not known whether the vibrations are produced incidentally or if they are addressed to specific receivers, who the receivers are, and what the actual meaning of the signal is.

We find that the repetition of temporal patterns in guarding vibrations is more variable, compared to foraging vibrations. We propose that the differences in variability encode the context, i.e., foraging or guarding. In the context of foraging, it is indicated that food source characteristics are encoded in pulse duration [5,65]. Whether a single temporal pattern of guarding vibrations might encode more detailed information on the cause of alarm remains to be answered.

So far, we can only speculate about the informational value of guarding vibrations.

The signal could be addressed to intruders, nestmates or both. From bumblebees, we know that hissing sounds are an aposematic warning signal directed to small vertebrates [28]. Whilst most stingless bee nests are well protected against small vertebrates, such as mice, some are regularly robbed by humans and apes that might be aposematically warned. However, it appears unlikely that guarding vibrations address them because the amplitude of the airborne components is very low and cannot be heard by the human observer. More likely is that the guarding vibrations are used to communicate with nestmates that could be informed about an intruder’s attack.

In social insect societies, individuals perform different tasks depending on their morphology, caste, sex, and age [66]. Depending on their duties, the meaning of signals and cues may be interpreted differently. Guarding vibrations and forager vibrations might cause different reactions depending on whether the recipient performs guarding or foraging tasks. When perceiving guarding vibrations, foragers and hive bees may stay inside the hive and stall the next foraging flight until the situation is cleared. Guarding bees in contrast could be motivated to fly out of the nest and attack the intruder. Similar behavioral patterns were found in mound-building termites, in which vibrations are used as an alarm signal. If a termite colony is attacked by an intruder, soldiers produce substrate vibrations. Vulnerable workers that sense these vibrations retreat into the nest while well-protected soldiers walk towards the source of the vibrations to defend the nest [67]. Vibrational alarm signals may have advantages over chemical alarm signals. Depending on the substrate, vibrational signals are transmitted faster than pheromones can spread in the nest atmosphere. Furthermore, the retention time of vibrations is much shorter compared to chemicals [68]. It is increasingly recognized that animal communication is complex and involves multiple sensory modalities. Vibrational signals are most likely one part of a multimodal information system used alongside chemical, tactile, and visual signals and cues.

In our study, the contexts of vibration production differed in a number of factors that should be controlled in future studies. Simultaneous recordings at different locations of the nest, playback experiments and testing of diverse stimuli will contribute to a better understanding of the vibrations. Further studies should address the multimodality of communication and experimentally study the factors inducing guarding vibrations. This will enable us to tell whether vibrations are incidentally produced, i.e., are cues or if they are produced with the intention of information transfer, i.e., serve as signals.

The muscle contractions of bees that cause vibrations inevitably come along with sound. Vibrations and nearfield sound could serve as information pathways between the sender and receiver. The range of signal transmission appears to differ considerably between these pathways. Based on behavioral observations and conclusions drawn from studies on honeybees, nearfield sound can only be perceived as air particle movements by bees standing very close to the vibrating forager, whereas substrate vibrations have a medium-range transmission [5]. We, therefore, consider the vibrational pathway more likely to be of importance compared to sound, i.e., air particle movements. However, future research has to investigate the sensory mechanisms underlying the perception of these signals in order not to rely on conclusions drawn from studies on other species.

## 5. Conclusions

Our experiments reveal that vibrations produced in the context of nest defense differ from those produced in the context of foraging and the arena experiments support the hypothesis that these vibrations are used in the context of nest defense and alarm communication.

## Figures and Tables

**Table 1:** Temporal patterns of foraging (nest) and guarding vibrations (entrance tube, arena) of *A. ferruginea.*

Recording Location	PSD [ms]			PD [ms]			IPD [ms]			DC			
	m	sd	*n*	m	sd	*n*	m	sd	*n*	m	sd	*n*	N
nest	214	±38	45	63	±8	51	149	±42	45	0.32	±0.09	45	7
entrance tube	766	±275	67	182	±45	76	582	±278	67	0.34	±0.11	67	9
arena	683	±287	398	267	±116	406	416	±307	398	0.47	±0.15	398	8

where PSD is pulse sequence duration, PD is pulse duration, IPD is inter-pulse duration, DC is duty cycle, m is mean, sd is standard deviation, *n* is total number of pulses, and N is number of analyzed pulse trains.

**Table 2:** Median coefficient of variation of thoracic vibration’s temporal structure per pulse train by *A. ferruginea foraging* (nest) and *guarding vibrations* (entrance tube, arena).

Recording Location	PSD		PD		IPD		DC		*n*
	CV	*p*	CV	*p*	CV	*p*	CV	*p*	
(1) nest	26.3 ^a^	(1–2) 0.009	23.6 ^a^	n.s.	35.0 ^a^	(1–2) 0.022	28.2 ^a^	(1–2) 0.011	7
(2) entrance tube	67.4 ^b^	(2–3) n.s.	38.3 ^a^		96.3 ^b^	(2–3) n.s.	54.4 ^b^	(2–3) n.s.	9
(3) arena	59.9 ^b^	(3–1) 0.017	61.8 ^a^		90.9 ^b^	(3–1) 0.009	39.6 ^a,b^	(3–1) n.s.	8

where PSD is pulse sequence duration, PD is pulse duration, IPD is inter-pulse duration, DC is duty cycle, superscript letters and *p* indicates statistical differences based on Kruskal-Wallis test and Dunn-Bonferroni-post hoc test (df = 2).

## Data Availability

The data presented in this study are available in article.
